# *In Vitro* Research Reproducibility: Keeping Up High Standards

**DOI:** 10.3389/fphar.2019.01484

**Published:** 2019-12-10

**Authors:** Cordula Hirsch, Stefan Schildknecht

**Affiliations:** ^1^Particles-Biology Interactions Laboratory, Swiss Federal Laboratories for Materials Science and Technology (Empa), St. Gallen, Switzerland; ^2^In vitro Toxicology and Biomedicine, Department of Biology, University of Konstanz, Konstanz, Germany

**Keywords:** cell culture models, reproducibility, toxicology, good cell culture practice, new approach methods (NAM)

## Abstract

Concern regarding the reproducibility of observations in life science research has emerged in recent years, particularly in view of unfavorable experiences with preclinical *in vivo* research. The use of cell-based systems has increasingly replaced *in vivo* research and the application of *in vitro* models enjoys an ever-growing popularity. To avoid repeating past mistakes, high standards of reproducibility and reliability must be established and maintained in the field of *in vitro* biomedical research. Detailed guidance documenting the appropriate handling of cells has been authored, but was received with quite disparate perception by different branches in biomedical research. In that regard, we intend to raise awareness of the reproducibility issue among scientists in all branches of contemporary life science research and their individual responsibility in this matter. We have herein compiled a selection of the most susceptible steps of everyday *in vitro* cell culture routines that have the potential to influence cell quality and recommend practices to minimize the likelihood of poor cell quality impairing reproducibility with modest investment of time and resources.

## Introduction

A survey published in Nature in 2016 (Baker, 2016) evaluating questionnaires on reproducibility in life science research disclosed not only the difficulties researchers have reproducing experiments from other laboratories, but also from their own. Even more surprising was the fact that awareness of this problem was widespread within the scientific community. The inability to reproduce study results, often inherent in observations from academic laboratories, are usually uncovered not without relevant delay, e.g. when potential therapies that are based on these findings transition from preclinical testing to the far more stringent conditions of clinical trials (Collins and Tabak, 2014). Needless to say, the societal costs associated with this problem are intolerable (Freedman et al., 2015). The controversial matter of insufficient reproducibility was, in fact, communicated openly in oncology and cardiovascular biology (Begley and Ellis, 2012; Errington et al., 2014; Libby, 2015). In toxicology, which may better reflect the background of most readers of this journal, awareness of this problem has emerged only gradually in association with insufficient *in vivo* reproducibility (Kilkenny et al., 2009; Voelkl et al., 2018). Such disclosures, in concert with studies indicating that *in vivo* data from rats and mice combined can only predict human clinical toxicology of less than 50% of candidate pharmaceuticals (Olson et al., 2000), promoted a revision of several toxicologists’ opinions towards mechanistic *in vitro* assays from the traditional reliance on pharmacological and toxicological *in vivo* animal testing.

## *In Vitro* Models in Life Science Research

A major concern raised by researchers in different fields of biomedicine was how a cell culture model, often not even originating from the organ of interest, could provide information about multilayer processes and pathological outcomes in humans. In this context, it is important to understand that application-oriented fields, such as pharmacology or toxicology, operate to a large extent on the fundamental progress made in biomedical research over the past decades and exploit the wealth of information generated about cellular stress pathways and molecular processes. This paradigm shift was largely shaped by the US National Research Council’s (NRC) strategic plan to modernize methods for testing environmental toxicants (Natl. Res. Counc., 2007). The approach envisions the identification of molecular targets and pathways that are linked with a toxicological outcome and fosters the establishment and validation of high-throughput new approach methods (NAM) for quantitative assessment of target perturbations (Collins et al., 2008; U.S. Environ. Prot. Agency, 2009). A key element in the NRC’s strategy is its distinct focus on the quantitative detection of perturbations of defined molecular events [Key Events, (KE)], cellular stress pathways, and marker signatures that are predictive for a specific outcome (Adeleye et al., 2015). The experimental *in vitro* model of choice, therefore, needs to express the pathway or mechanism of interest and also needs to allow quantitative determination of the disturbance caused by the stressors. In this regard, the concept of adverse outcome pathways (AOP) was designed as a conceptual framework for the sequential organization of the molecular initiating event (MIE), connected with the adverse outcome (AO) *via* a series of KEs (Ankley et al., 2010). The AOP concept fosters the development or selection of assays allowing a quantitative detection of individual KEs, thereby enabling the definition of threshold levels (Leist et al., 2017; Terron et al., 2018). AOP also represents an organizational tool for identification of additive or synergistic effects that might occur through activation of identical or different KEs by two or more compounds. The stringent demand for precise quantitative and qualitative information required for AOPs illustrates the explicit necessity for experimental models with a high rate of reproducibility and the necessity for increased awareness of the reproducibility problem in all branches of life science research.

## Insufficient Reproducibility in Cell Models

A defined assay performed with a defined *in vitro* model needs to yield identical results— no matter when or where it is performed. As trivial as this statement may appear, its implementation is quite difficult in reality. The Nature survey of 2016 (Baker, 2016) highlighted the degree of inadequate reproducibility in biomedical research and underlined the widespread awareness of the problem within the scientific community. It is, thus, all the more astonishing that systematic comparisons of experimental models applied in different laboratories are rather rare, particularly in the field of *in vitro* research. In nanotoxicology, *in vitro* toxicity assays are the most frequently used approaches to assess potential hazardous effects of engineered nanomaterials (Guggenheim et al., 2018). This is mainly due to the fact that researchers early on realized that the immense number of newly developed nanomaterials would make it impossible to perform classical *in vivo* animal tests due to the amount of time, money, and number of animals required (Hartung and Sabbioni, 2011; Schrurs and Lison, 2012; Guggenheim et al., 2018). Nanomaterials exhibit unique properties due to their small size that make them suitable for many different applications. However, these same particle properties often interfere with experimental test systems (Wörle-Knirsch et al., 2006; Laurent et al., 2012; Bohmer et al., 2018). Insufficient nanoparticle characterization, unidentified interference with test systems, and poor definition of controls for monitoring assay performance led to several contradictory observations in the early days of nanotoxicology research (Hirsch et al., 2011). However, this shaky start allowed nanoparticle toxicology to emerge as one of the fields in which the aspect of adequate reproducibility of *in vitro* studies gained appropriate attention, and consequently, allowed an open discussion of this topic. An exemplary illustration of this transparency is a publication by Elliott and colleagues (Elliott et al., 2017) assessing the reproducibility of MTS-tetrazolium reduction assay results as indicators of cell viability in an international inter-laboratory comparison study with five independent laboratories. Strict standard operating procedures (SOP) were employed using a sophisticated 96-well plate design that allowed detection of up to seven parameters of assay performance, including accuracy of multi-channel pipetting, cell handling/cell growth, and instrument performance (i.e. plate reader issues) (Rösslein et al., 2015). A549 cells were purchased from two independent, credible, accepted commercial sources and both, seemingly identical, cell cultures were used in all labs. Even under such strict conditions, EC_50_ values of the two A549 cultures upon CdSO_4_ treatment differed by a factor of two in all laboratories. In the course of these investigations, cell line authentication was discovered to be one of the main factors influencing assay results. Short tandem repeat sequencing revealed a partial chromosome deletion in one of the cell cultures. Technical aspects also contributed to result variability. For example, simple cell handling steps, such as PBS washing, were identified to significantly change assay outcomes. This example provides a vivid illustration of the impact of seemingly trivial details and the necessity to draw attention to all aspects of *in vitro* experimentation.

A recent evocative study of the mammary epithelial cell line MCF10A and growth rate inhibition by anti-cancer drugs systematically addressed inter- and intra-study center variations and identified factors contributing to insufficient reproducibility (Niepel et al., 2019). Although the five research centers applied cells and chemicals of the same stock, astonishing center-to-center variations up to 200-fold were observed in growth inhibition rates. Cell seeding, i.e. slight variations in initial cell numbers, was identified as one key source of these variations (for more details see Recommendation *5) (Cell density and medium change).* Overall, the subtle interplay between experimental methods and a vast array of poorly defined sources of biological variability was found to be the main cause of the observed irreproducibility. For example, two distinct methods were used to quantify cell viability: *a)* microscopic cell counting as a direct measure of viable cell number and *b)* detection of intracellular ATP levels as a proxy of viable cells. ATP levels do not necessarily directly correlate to the number of viable cells, resulting in identical EC_50_ values for some drugs, but differing greatly for others. Changes in ATP levels following treatment could be the consequence of cell death, effects on cell proliferation or the alteration of cellular ATP metabolism. Furthermore, linearity between cellular ATP levels and cell viability is not justified for many cell types. In several cases, a reduction of ATP levels by almost 50% is tolerated by cells without significant influence on cell viability (Pöltl et al., 2012). In conclusion, while both assays (direct cell counting and ATP measurements) might be quite robust and reproducible *per se*, they provide different information from their results, e.g. drugs that alter cellular ATP metabolism, and are thus not interchangeable in these cases. As a consequence of the huge number of individual biological factors involved, Niepel and colleagues came to the rather discouraging conclusion that “most examples of irreproducibility are themselves irreproducible*”* (Niepel et al., 2019).

This spectrum of biological factors further depends on the complexity of the cell model applied. The introduction of 2D co-culture models and 3D cell models was motivated by the ambition to recapitulate the natural *in vivo* environment of cells in a cell culture dish. In fact, cells in a 3D culture differ morphologically and physiologically from their counterparts in a 2D setup (Baharvand et al., 2006; Edmondson et al., 2014). Introduction of the third dimension in a cell culture model results in additional parameters that could potentially affect reproducibility, including spheroid size and consequently the oxygen and nutrient supplies to cells in different layers within the structure; spatial organization of surface receptors involved in interactions with neighboring cells; activation of signal transduction pathways; and induction of gene expression profiles (Vinci et al., 2012). All of these changes ultimately have the potential to influence cell biology and cellular response towards exogenous stressors. These aspects were exemplified by a study using HCT-116 cells in 3D culture that displayed increased resistance against anti-cancer drugs compared with the 2D model (Karlsson et al., 2012). The results in the 3D model more closely reflected *in vivo* observations. Nonetheless, the initial euphoria regarding such studies became gradually overshadowed by higher rates of insufficient reproducibility observed in complex cell models. To our knowledge, no systematic comparison of the reproducibility of cells in mono-culture vs. their integration into more complex models (co-culture, 3D, etc.) has yet been published. Rumors from the industry, however, indicate a returning trend towards more elementary cell models with robust readouts that allow both adequate predictivity and high reproducibility. This does not mean that complex cell models are inappropriate with respect to their reproducibility *per se*, but they require far higher investment in their characterization and validation to limit the degree of overall variability compared with less complex models.

## Good Cell Culture Practice: Suggestions to Improve Reproducibility

**Figure 1:**
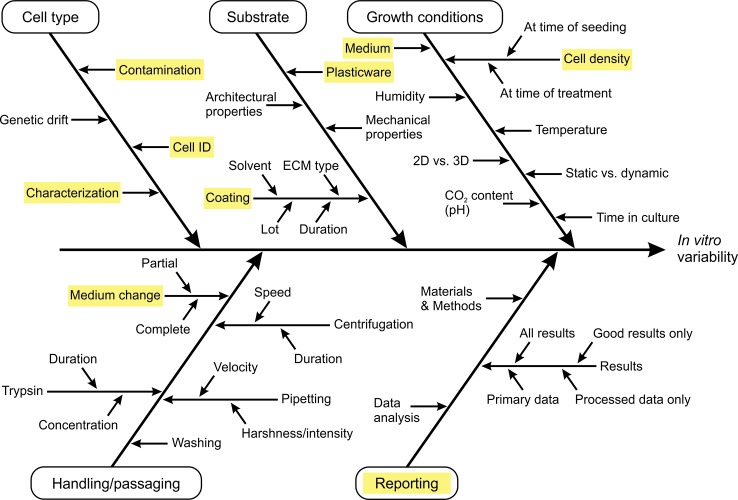
Cause-and-effect diagram summarizing potential sources of variability relevant for *in vitro* assays. According to Petersen et al. (2019) cause-and-effect analysis was applied to visualize sources of variability. We do not claim completeness of the information but rather encourage researchers to complement and/or adapt the diagram for their specific cellular model or field of research and take the provided information as a starting point to challenge and scrutinize their own working standards. Keywords marked in yellow are discussed specifically in the main text.

To the best of our knowledge, no specific field of *in vitro* research faces greater issues of reproducibility than another. No particular cell line, cellular model, or particular assay seems to be favorable in this regard. In contrast, all fields of *in vitro* toxicology seem to face certain— though not necessarily the same— issues of irreproducibility. Therefore, the question arises which elements in *in vitro* biomedical research are potential sources of unsatisfactory reproducibility, and can be actively influenced by individual researchers with manageable effort and within the framework of the existing scientific system. Over the course of the past two decades, initiatives to improve the quality of *in vitro* research have identified critical aspects of *in vitro* cell culture routines and their influence on reproducibility. The concept of *Good Cell Culture Practice* (GCCP) (Coecke et al., 2005) was developed and gradually adapted to ongoing scientific progress (Pamies et al., 2018) as a guidance document for *in vitro* reporting standards. Other initiatives, such as the OECD guidance document for *Good In Vitro Method Practices* (GIVIMP) (Eskes et al., 2017), defined standards for regulatory testing under *Good Laboratory Practice* (GLP) rules. Recently, Petersen and colleagues very specifically discussed sources of variability in four distinct nano-bioassays (Petersen et al., 2019). The following selection of approaches to improve reproducibility of *in vitro* studies was loosely inspired by these initiatives and makes no claim to completeness. It is instead based on the experiences of the authors and communications with colleagues from adjacent scientific disciplines. An explicit emphasis was placed on broadly applicable techniques to improve the reproducibility of results obtained with cellular *in vitro* models, which can be implemented with relatively little investment and provide major benefits for the individual project and the scientific community as a whole. **Figure 1** provides a summary of potential sources of variability that might influence *in vitro* results. The particular aspects marked in yellow in the diagram are discussed in more detail.

## Recommendations

1. *Selection of plasticware*: Cell culture flasks and dishes are manufactured from different materials, such as tissue culture polystyrene or polyethylene terephthalate (PET). Each of these materials possesses different surface properties that affect the interaction of cells with the plastic surface. Even identical formats of cell culture dishes and plates made from the same type of plastic by different manufacturers can have different surface properties as a consequence of production variability (e.g. temperature, duration of the production process, or supplier of raw materials) (Battiston et al., 2012). Surface parameters, such as hydrophilicity or electrical charge, can influence attachment and activation states of cells. It has been shown that monocyte adhesion, cytokine release, maturation of oocytes, and differentiation of neurons, to name just a few, can be significantly affected by different types of plastic and different plastic manufacturing processes (Shen and Horbett, 2001; Brodbeck et al., 2002; Clinchy et al., 2003; Schildknecht et al., 2009). These examples clearly illustrate the fundamental importance of an initial characterization of the influence of the plasticware used for the cell model and the importance of adequately reporting materials.
2. *Coating*: Cells embedded within a tissue interact with the extracellular matrix to develop cell type-dependent features such as morphology, function, proliferation, differentiation, gene expression pattern, and even survival (Dike and Farmer, 1988; Longhurst and Jennings, 1998; Damsky and Ilić, 2002). Likewise, coating of cell culture plastic with extracellular matrix proteins such as collagen, fibronectin, or laminin offers specific contact partners for interaction with adhesion receptors (e.g. integrins) on the cell surface (Giancotti and Ruoslahti, 1999). Certain cell types strongly depend on an appropriate coating and absence of extracellular matrix components can even lead to the complete demise of the affected cells. Obviously, in specific fields of *in vitro* research, e.g. stem cell differentiation, the coating receives a great degree of attention (Somaiah et al., 2015; Abdal Dayem et al., 2018; Sun et al., 2018). This is in contrast to more conventional and easy-to-handle tumor or immortalized cell lines, which seem to grow on any kind of bare cell culture plastic. However, the extracellular matrix has also been shown to influence adhesion, proliferation, mobility, and morphology of rather robust cell lines (Fiegel et al., 2004; García-Parra et al., 2013; Liberio et al., 2014). For example, in the field of neuroscience, adhesion and neurite outgrowth of the pheochromocytoma cell line PC12 are highly dependent on the coating composition and coating procedures applied (Orlowska et al., 2017; Teppola et al., 2018). Similarly, different coatings reportedly influence attachment, nuclei shape, branching of neurites and neuronal network formation of the neuroblastoma cell line SH-SY5Y. The hepatoma cell line cell line HepG2, which is widely used not only for liver-associated research, seeded on polystyrene well plates with and without coating exhibited significant differences in their morphology, distribution, and functions such as particle uptake or P450-dependent detoxification (Saravia and Toca-Herrera, 2009; Prats-Mateu et al., 2014). As a large percentage of biomedical research is actually conducted with such immortal, presumably easy-to-handle cell lines, an explicit focus on the precise documentation of coating details (i.e., coating components, amount, volume of coating per well, coating period, washing steps, storage after coating) is essential to ensure reproducibility.
3. *Cell characterization*: A large percentage of research is conducted with a relatively small number of tumor or immortalized cell lines. Unfortunately, there is a clear trend that the more unpretentious and widely distributed the cell line, the less emphasis is spent on its characterization. Long-term sub-culturing, for instance, puts selective pressure on individual cells in the culture with higher growth rates. After a few passages, this can lead to complete exclusion of slower proliferators and may culminate in genetic or phenotypic alterations, e.g. accumulation of mutations or changes in morphology, development, or gene expression patterns (Hughes et al., 2007). Systematic investigations revealed significant genetic differences in widely applied cell models between different laboratories (Frattini et al., 2015; Kleensang et al., 2016; Gutbier et al., 2018). Furthermore, contamination of cell lines with other cells is a frequent problem in *in vitro* research and can only be excluded by elaborate genetic authentication. Additionally, cell lines are often distributed by informal exchange between laboratories, a process that is far less often accompanied by a transfer of relevant information regarding cell passage number or origin of the cell line, let alone genetic characterization. In order to limit these potential sources of error, cell lines should be acquired only from established (commercial and non-commercial) cell culture banks (e.g. ATCC, ECACC, DSMZ, JCRB, CellBank Australia) that guarantee an examination of cross-contamination as well as molecular cell authentication on the basis of fingerprinting, single locus short tandem repeats (STR) and single nucleotide polymorphism (SNP) profiling (Capes-Davis et al., 2010; Didion et al., 2014). Establishment of a new cell culture from a single vial, in general, represents a bottleneck situation with the potential to select slightly modified "subclones" in the cell population and hence, deserves particular attention by researchers. Upon arrival and initial thawing of thoroughly characterized cells, a large stock of vials (> 100 vials) should be prepared. This not only guarantees availability of cells with a relatively low passage number over years and decades, but also allows assessment of the potential influence of freezing/thawing by comparison of the initially received cells with cells that have undergone a freezing/thawing cycle.
4. *Selection of cell culture media*: Once a cell line is introduced in a laboratory, little emphasis is often placed on the cell culture medium. Approximately 90% of all reported *in vitro* experiments are currently conducted with one of four different types of medium: DMEM, RPMI, M199; MEM (McKee and Komarova, 2017). The fact that the electrolyte composition and carbohydrate content of these types of medium are usually not ideal physiological environments is often overlooked. A typical scenario resulting from the use of these media is an oversupply of phosphate and/or a limitation of calcium and magnesium (McKee and Komarova, 2017). Typical plasma glucose levels are in the range of ca. 5 mM in healthy humans, yet many commonly used media contain 25 mM glucose. Such conditions support reliance on glycolysis and therefore increase independence from mitochondrial energy supplies. Maintenance of a cell model in either low or high glucose conditions is therefore likely to influence experimental treatments that interfere with energy metabolism. Another often ignored factor is the gender of the cell donor (De Souza Santos et al., 2018). Estrogen receptors are found on most cell types and exposure of male cells to estrogenic media (e.g. phenol red structurally resembles some nonsteroidal estrogens) can lead to estrogen-receptor-dependent influences on cell proliferation, differentiation, and metabolism (Berthois et al., 1986; Farzaneh and Zarghi, 2016). In female cells, culture conditions can lead to a release of the epigenetic inactivation of one of the two X-chromosomes and, consequently, the expression of gene products from both X-chromosomes, coding for a large number of genes involved in metabolism and general cell function (Shah et al., 2013). Cell culture media in use today inevitably creates conditions that are not directly comparable to conditions *in vivo*. An awareness of the constituents of the medium used and their influence on cellular processes are cornerstones of improved reproducibility. There is no right or wrong medium or cell model, but to ensure reproducibility, general cell culture parameters need to be fixed, and more importantly, the experimenter needs to be aware of the features, strengths and weaknesses of the *in vitro* model when interpreting the data it generates.
5. *Cell density and medium change*: After identification of the so-called “inoculum effect,*”* which describes increases in the minimal inhibitor concentration of an antibiotic with increased numbers of bacterial cells, it soon became apparent that the density of eukaryotic cells can have a profound influence on the outcome of toxicological studies (Ohnuma et al., 1986; Brook, 1989; Schildknecht et al., 2009; Schildknecht et al., 2011; Scholz et al., 2011). In addition to the statistical decrease in drug accumulation (Takemura et al., 1991; Schildknecht et al., 2015), higher cell densities are characterized by more pronounced paracrine signaling and contact inhibition. These events can influence cell metabolism, which in turn modulates cellular responses to pharmaceutical or toxicological compounds, and, consequently, cell viability. It is therefore apparent that modest variations in the number of seeded cells, e.g. as a consequence of differences in counting methods, can lead to significant disparities in cell density after a few days of proliferation. High cell densities also influence the composition of media constituents and the accumulation of waste products, such as lactate or ammonia. All of these parameters can be manipulated by the frequency of medium changes (Wright Muelas et al., 2018). Medium changes have been shown to influence oxygen supply and cellular metabolism, as well as synthesis of proteins, DNA, and RNA (Al-Ani et al., 2018; Wright Muelas et al., 2018). These observations clearly highlight the necessity for precise documentation and reporting of parameters like cell density and frequency of medium changes to minimize their contribution to insufficient reproducibility.
6. *Mycoplasma contamination*: Receipt of (often non-tested) cells from other laboratories bears the threat of contamination by mycoplasma or viruses, which can cause a series of effects in the infected cells. For a long time, the most reliable test to identify mycoplasma contamination involved their cultivation and detection by microbiological assays, requiring a testing period of about one month (Rottem and Barile, 1993). These prolonged tests have been largely replaced by a PCR-based technique that has emerged as the method of choice for fast and reliable detection of mycoplasma (Nübling et al., 2015). To avoid mycoplasma infections, the best way to handle newly arrived cells is to thaw and cultivate them in quarantine until the testing results arrive. It is also important to strictly avoid storage of non-tested cells in liquid nitrogen tanks where there is potential for direct physical contact between the cells and the nitrogen. Furthermore, routine testing of applied cell lines should be conducted several times per year to ensure early detection of contamination that might occur in everyday routine work. The introduction of a fast and reliable test for mycoplasma detection has significantly lowered the burden of contamination inspections. Hence, mycoplasma testing should be a mandatory element in any cell culture laboratory.
7. *Reporting:* An obvious reason for poor reproducibility is insufficient, incomplete, and inaccurate reporting of methodologies. Although detailed guidelines on GCCP and reporting standards have been published, a brief glimpse at the materials and methods section of submitted and published manuscripts clearly reveals that there is still a long and difficult road ahead. Far too often, the materials and methods section becomes the first casualty when journal word count limits urge authors to scale down manuscripts. On the other hand, it is commonly accepted that the quality of reporting methodologies ranks among the most important elements for enhancing reproducibility. In plain words, good reporting covers all the information another researcher needs to exactly reproduce an experiment. Initiatives fostering a standardization of documentation required for cell models culminated in the definition of GCCP standards (Coecke et al., 2005; Hartung et al., 2019), which were recently modified to meet requirements that have emerged with the advent of stem cell research (Pamies et al., 2017). GCCP guidance focuses on the following principles: *1)* understanding the *in vitro* model; *2)* quality of materials and methods; *3)* documentation of information necessary to allow repetition of the work; *4)* protection of the environment and individuals from hazards; *5)* compliance with laws, regulations, and ethical principles; and *6)* training of staff to ensure quality work and safety. The primary focus of the GCCP principles is documentation of the test models, and initiatives to standardize method documentation have resulted in the introduction of SOPs. Currently, there is no obligatory organization of SOPs in the field of *in vitro* toxicology. Initiatives such as the EURL ECVAM DataBase service on Alternative Methods to Animal Experimentation (https://ecvam-dbalm.jrc.ec.europa.eu) have compiled a large and steadily growing collection of protocols organized in a standardized and uniform manner. Depending on the acceptance and distribution of these protocols within the scientific community, their organization could serve as a solid platform for the generation of new SOPs relevant to *in vitro* research in the future. The formulation and application of SOPs is undoubtedly a central element to improving experimental reproducibility. Thoughtless use of SOPs, however, could lead to insufficient integration of new knowledge into existing methods and must be guarded against. Reproducibility is a central aspect of life science research, but it must not prevent progress. Centralized collections of SOPs and methods need to guarantee precise documentation of high quality methods but should also allow modifications of protocols through a structured process of discussion and consensus among experts in respective fields to ensure both innovation and replication. Appropriate and complete reporting, however, does not end at the raw data level. Data handling further influences result reproducibility. For example, the use of either absolute or normalized data is often not adequately indicated in study descriptions. Normalization usually starts with definition of control values as 100% and all other data are consequently indicated as percentages of the control values. The inclusion and definition of 0% values is however often ignored, but is equally relevant, e.g. for the calculation of IC_50_ values. In cell-based toxicological investigations, sigmoidal curves are usually obtained by a four-parameter fit (lower and upper asymptotes, turning point, and steepness of the curve at the turning point). Automatic fitting often leads to curves that fail to hit the 100% bar due to problems with control values. In such cases, re-normalization procedures are required, e.g. to determine benchmark responses, and 2–3 points in the no-effect range could alternatively be applied for re-normalization (Krebs et al., 2018). Such interventions, however, can only be justified on the basis of a researcher’s profound knowledge of the biology of the experimental model and the test assay applied. Krebs and colleagues recently published an annotated template for the description of cell-based toxicological test methods, based on the OECD guidance document 211 (GD211) (Krebs et al., 2019). The template provides a clearly defined structure intended to support researchers in the design of their study and in handling of the data obtained. It has been explicitly compiled in user-oriented fashion to gain greater acceptance by the research community. Finally, the practice of "reporting the best results" only and ignoring those experiments that failed to yield the expected outcome has an impact on data reproducibility. Some journals already request the publication of all raw data, e.g. displaying entire western blots instead of cut-out bands of interest. This aspiration could be amended by the publication of all experiments performed to address a defined question, even those that are not included in the main body of a manuscript. Whether this attempt to include all data would actually be implemented by researchers and publishers is a whole other question.

## Discussion and Outlook

The necessity for enhancement of result reproducibility in the life sciences has been identified and is gradually being internalized by researchers and institutions alike (Prinz et al., 2011; Drucker, 2016). Achieving high rates of reproducibility often stands in contradiction to the discovery of novel scientific insights. Although it is obvious that new findings are only of use if they can be reproduced, Dirnagel recently cautioned that cutting-edge discovery is unavoidably associated with a high rate of false positive results (Dirnagl, 2019). These false positive results are an integral part of exploration and must not be conflated with intentional manipulation of results.

Even with the best of intentions, it must be concluded that the limits of reproducibility in cell culture work is reached when confronted with the question of reference standards, particularly for established and widely distributed cell lines. Simply put, which of the currently available and characterized stocks of common cell lines, like HeLa cells, should be considered as the gold standard? Even if a consensus could be reached for individual cell lines, storage capacity limitations force even large cell banks to passage their cells, which necessarily influences the cells in one way or another over time.

We have written the present article as a condensed introduction of effective, easy-to-implement measures to improve reproducibility of experimental results. In plain summary, the most relevant rules are:

- Ensure an in-depth understanding of the cell model by the researchers working with it; a thorough characterization is a prerequisite to identify the validity of a cell model and its limits.
- Pay attention to seemingly irrelevant details; the selection of plasticware, coating, or the type of medium, can have significant influence on the outcome of a study as summarized in **Figure 1**.
- Be aware of what an experimental readout actually detects. Viability, for instance, is routinely assessed by different assays (e.g. resazurin reduction, LDH release, ATP detection) but they measure different cellular parameters and hence cannot be compared directly.
- Provide all the information in your reporting that is necessary to precisely reproduce your experiment.

Above all the aspects discussed, one of the most influential factors in any attempt to improve reproducibility is a researcher’s consolidated knowledge about the cell model in use (see Principle 1 of GCCP; Coecke et al., 2005). The in-depth characterization of cellular parameters relevant to a given scientific question is certainly a resource-consuming endeavor, but is a worthwhile investment in the long run, both in the selection of an appropriate cell model and interpretation of the results. As the residence time of scientists in laboratories is often limited, the quality and continuity of their supervision by experienced staff becomes a critical factor in knowledge transfer. The second of the most influential factors contributing to insufficient reproducibility is the lack of generally accepted and mandatory guidelines on the cultivation of individual cell lines. Guidance documents such as the GCCP or GIVIMP, have been published for quite some time, but so far have not gained the attention they deserve from researchers and publishers alike. The question therefore arises how consensus on a standard protocol for a given cell line can be achieved and how its application can be motivated. This task can only be accomplished by members of the communities regularly using a given cell line. The formulation of new guideline protocols should be fostered by the respective scientific societies. Well established and influential laboratories might play a key role to reach consensus on a ready-to-use standard protocol for the handling of a given cell line. Application of these protocols should in a next step become mandatory for the acceptance of a new study by the scientific community, unless deviations from the standard protocol can be scientifically justified. Such measures will not bring overwhelming scientific merit for the individual scientist, but are inevitable steps to re-establish and maintain confidence of both researchers and the general public in contemporary biomedical *in vitro* research.

## Author Contributions

CH and SS contributed equally to the preparation of the manuscript.

## Funding

We acknowledge funding from the NanoScreen Materials Challenge co-funded by the Competence Centre for Materials Science and Technology (CCMX) as well as support by the Doerenkamp-Zbinden foundation, the Land-BW (N EURODEG), the BMBF (NeuriTox) and by the Projects from the European Union’s Horizon 2020 research and innovation program EU-ToxRisk (grant agreement No 681002).

## Conflict of Interest

The authors declare that the research was conducted in the absence of any commercial or financial relationships that could be construed as a potential conflict of interest.
